# Liposomal bupivacaine intercostal nerve block for pain control in thoracoscopic surgery: a randomized controlled trial

**DOI:** 10.3389/fmed.2025.1647324

**Published:** 2025-09-04

**Authors:** Yuqing Chi, Xiaoqun Su, Shuxian Liu, Junhua Wen, Maoyan Hu, Haonan Li, Hualing Huang, Zhijing Zhang, Haihui Xie

**Affiliations:** ^1^Shenzhen School of Clinical Medicine, Southern Medical University, Shenzhen, China; ^2^Department of Anesthesiology, The Tenth Affiliated Hospital, Southern Medical University (Dongguan People’s Hospital), Dongguan, China; ^3^Department of Gynaecology and Obstetrics, The Tenth Affiliated Hospital, Southern Medical University (Dongguan People’s Hospital), Dongguan, China; ^4^Dongguan Key Laboratory of Anesthesia and Organ Protection, Dongguan, China

**Keywords:** liposomal bupivacaine, bupivacaine hydrochloride, thoracic surgery, postoperative analgesia, intercostal nerve block

## Abstract

**Objective:**

This study aimed to evaluate and compare the analgesic efficacy of liposomal bupivacaine (LB) versus conventional bupivacaine hydrochloride for intercostal nerve block after thoracoscopic surgery.

**Design:**

A prospective, randomized, controlled, single-blind study.

**Setting:**

The study was conducted in the operating room, post-anesthesia care unit (PACU), and general ward.

**Participants:**

A total of 100 patients classified as ASA physical status II–III who were scheduled for thoracoscopic surgery were enrolled.

**Interventions:**

Participants were randomly allocated to receive either LB or conventional bupivacaine hydrochloride via intercostal nerve block, performed under ultrasound guidance. All patients received intravenous patient-controlled analgesia (PCA) without a continuous background infusion. Rescue morphine was administered as needed if the PCA failed to provide adequate pain relief (VAS ≥ 4).

**Measurements:**

The primary outcome was postoperative pain intensity assessed using the Visual Analog Scale (VAS; 0–10) both at rest and during exercise at 6, 8, 12, 24, 48, and 72 h after surgery. Secondary outcomes included total morphine consumption, PCA demand frequency, patient satisfaction scores, intraoperative remifentanil dose, and length of hospital stay. Safety outcomes included the incidence of postoperative nausea and vomiting (PONV), pruritus, pulmonary complications, and cardiovascular events.

**Results:**

Baseline characteristics and surgical procedures were comparable between groups. Compared with conventional bupivacaine, the LB group showed significantly lower VAS scores at rest and during exercise at all six postoperative time points (6–72 h; all *p* < 0.01). PCA demand frequency was significantly reduced in the LB group (median: 11 vs. 30 presses; *p* < 0.01). Patient satisfaction scores were significantly higher in the LB group (median: 9.0 vs. 7.0; *p* < 0.01). No significant differences were observed in intraoperative remifentanil consumption (*p* = 0.088) or postoperative hospital stay (*p* = 0.135). Rescue morphine requirements were minimal in both groups (median: 0 doses).

**Conclusion:**

LB provided sustained and effective postoperative analgesia for 72 h after thoracoscopic surgery, while significantly reducing opioid consumption (*p* < 0.01) and supplemental analgesic requirements compared to conventional bupivacaine.

**Clinical trial registration:**

www.chictr.org.cn, ChiCTR2300076708.

## 1 Introduction

Perioperative multimodal analgesia (PMA) is currently recognized as the gold standard in pain management. This approach strategically combines multiple analgesic modalities to achieve optimal pain control while minimizing adverse effects, reducing opioid-related complications, and maintaining physiological homeostasis (1). Local anesthetics and peripheral nerve blocks are indispensable elements within a PMA regimen, which contribute to reducing opioid consumption and minimizing the associated side effects (2–4). However, their clinical utility is limited by the transient nature of analgesia, typically lasting only several hours post-administration (5). While continuous nerve block techniques can prolong analgesia, they carry inherent risks including catheter-related infections, hematoma formation, nerve injury, and catheter displacement (6). Liposomal bupivacaine (LB) has emerged as an innovative solution within this paradigm, providing sustained analgesia (up to 72 h) from a single administration. This extended-duration formulation significantly improves postoperative pain management while reducing opioid dependence (7–9).

The persistently high incidence of lung cancer and pulmonary nodules has driven a steady annual increase in thoracic surgery procedures (10). Given that these operations typically induce substantial postoperative pain, optimal analgesia is critical for recovery (11), and prevention of post-thoracotomy pain syndrome (12). Intercostal nerve blockade has gained prominence in thoracoscopic surgery due to its demonstrated efficacy in acute pain management, combined with technical safety and procedural simplicity (13). However, the optimal local anesthetic choice for this block remains controversial. Furthermore, existing evidence regarding liposomal bupivacaine’s effectiveness in this specific application remains inconclusive.

We hypothesized that LB would extend the duration of intercostal nerve blockade and improve analgesic efficacy compared to conventional bupivacaine. To test this hypothesis, we conducted a randomized controlled trial evaluating the combined use of LB and intercostal nerve block for post-thoracoscopic analgesia.

## 2 Materials and methods

### 2.1 Study population and randomization

This randomized controlled trial received ethical approval (KYKY2023-034; September 13, 2023) from the Institutional Review Board of our institution. Written informed consent was obtained from all participants before enrollment. The trial was prospectively registered at the China Clinical Trials Registry (ChiCTR2300076708; October 17, 2023; www.chictr.org.cn), complying with ICMJE clinical trial registration requirements.

Participants were recruited from November 2, 2023 to September 26, 2024 at the Tenth Affiliated Hospital of Southern Medical University. Inclusion criteria were: (1) age 18–65 years (based on bupivacaine safety considerations in elderly patients); (2) BMI 18–30 kg/m^2^; (3) ASA physical status II-III; and (4) scheduled for elective thoracoscopic surgery. Exclusion criteria comprised: (1) known hypersensitivity to local anesthetics; (2) pre-existing neurological disorders; (3) chronic opioid use; (4) refractory hypotension (SBP < 90 mmHg); and (5) significant cardiac disease (including severe arrhythmias or advanced heart block).

Following written informed consent, participants were randomly allocated to receive either LB or conventional bupivacaine hydrochloride in a 1:1 ratio through block randomization. The allocation sequence was computer-generated using SPSS software (version 25.0; IBM Corp).

### 2.2 Blind procedures

This study employed a single-blind design where: patients remained blinded to treatment allocation throughout the study period and the investigating anesthesiologist and statistician were unblinded.

### 2.3 Sample size calculation

**Figure 1:**
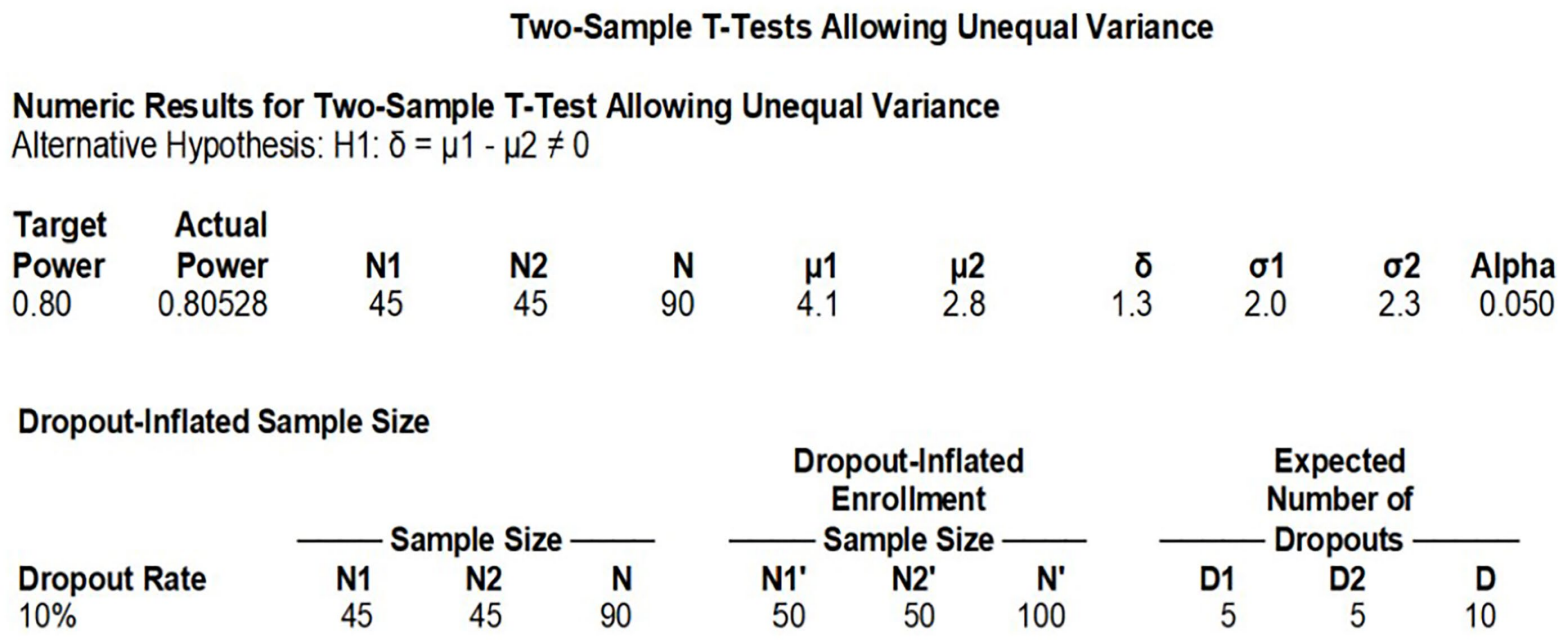
Sample size calculation.

This randomized controlled trial compared LB versus conventional bupivacaine hydrochloride, with the primary endpoint being exercise-induced VAS pain scores at 72 h postoperatively. Based on prior data from Kenneth et al. (14) (control group: 4.07 ± 2.0 vs. experimental group: 2.79 ± 2.25), we performed an *a priori* power analysis using the following parameters: Two-tailed *α* = 0.05, Power (1-*β*) = 80%, Effect size (d) = 0.65 (calculated from mean/SD differences). The analysis indicated a requirement of 45 patients per group (*N* = 90 total). Accounting for a 10% attrition rate, we planned to enroll 50 participants per group (total *N* = 100) to ensure adequate statistical power (See Figure 1).

### 2.4 Anesthesia protocol

All patients received standardized monitoring comprising continuous electrocardiography, pulse oximetry, and non-invasive blood pressure measurement. General anesthesia was induced with intravenous sulfentanyl (0.5 μg/kg), etomidate (0.2 mg/kg), and cisatracurium (0.2 mg/kg). Maintenance was achieved through continuous infusion of remifentanil (0.1–0.5 μg/kg/min) and cisatracurium (1–2 μg/kg/min), with dosages titrated according to hemodynamic responses and bispectral index (BIS) values (target range 40–60). Following induction, invasive monitoring was established via radial arterial catheterization for continuous blood pressure measurement and central venous cannulation through the internal jugular vein. Mechanical ventilation was initiated with volume-controlled mode, maintaining tidal volumes of 6–8 mL/kg (double-lung ventilation) or 4–6 mL/kg (single-lung ventilation). Respiratory rates were adjusted to target end-tidal CO₂ of 35–45 mmHg while ensuring SpO₂ > 92%. Positive end-expiratory pressure (PEEP) of 5 cmH₂O was applied throughout the procedure.

### 2.5 Study interventions

All participants underwent standardized thoracoscopic procedures performed by the same surgical team, with identical port placement, incision numbers, and chest tube positioning. For regional analgesia:

LB group: Received 266 mg LB diluted in 20 mL normal saline (13.3 mg/mL concentration), with 4 mL (53.2 mg) administered per rib under ultrasound guidance. Control group: Received 37.5 mg conventional bupivacaine hydrochloride in 15 mL normal saline (2.5 mg/mL concentration), with 3 mL (7.5 mg) injected per rib.

**Figure 2:**
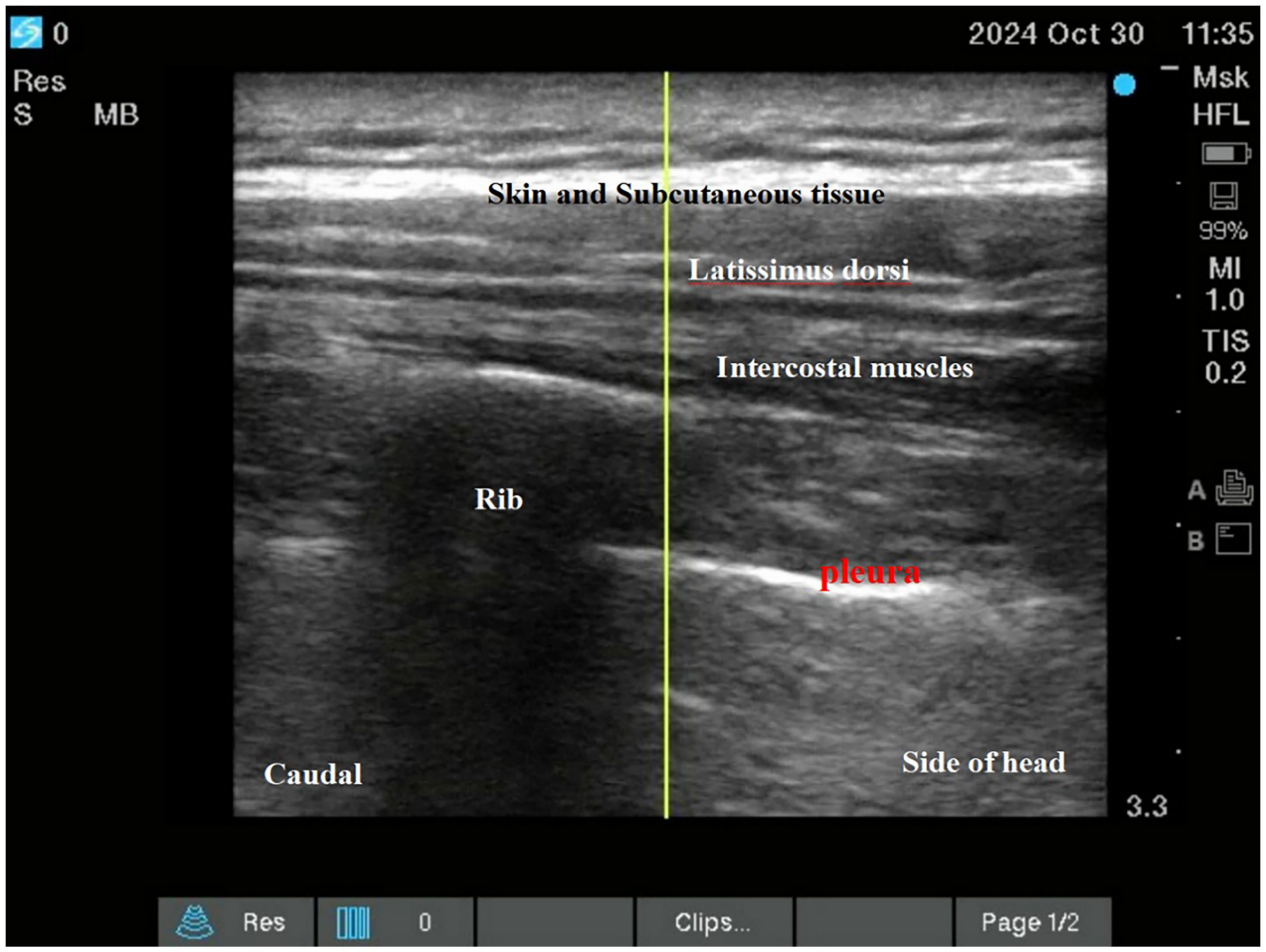
Intercostal nerve block.

**Figure 3:**
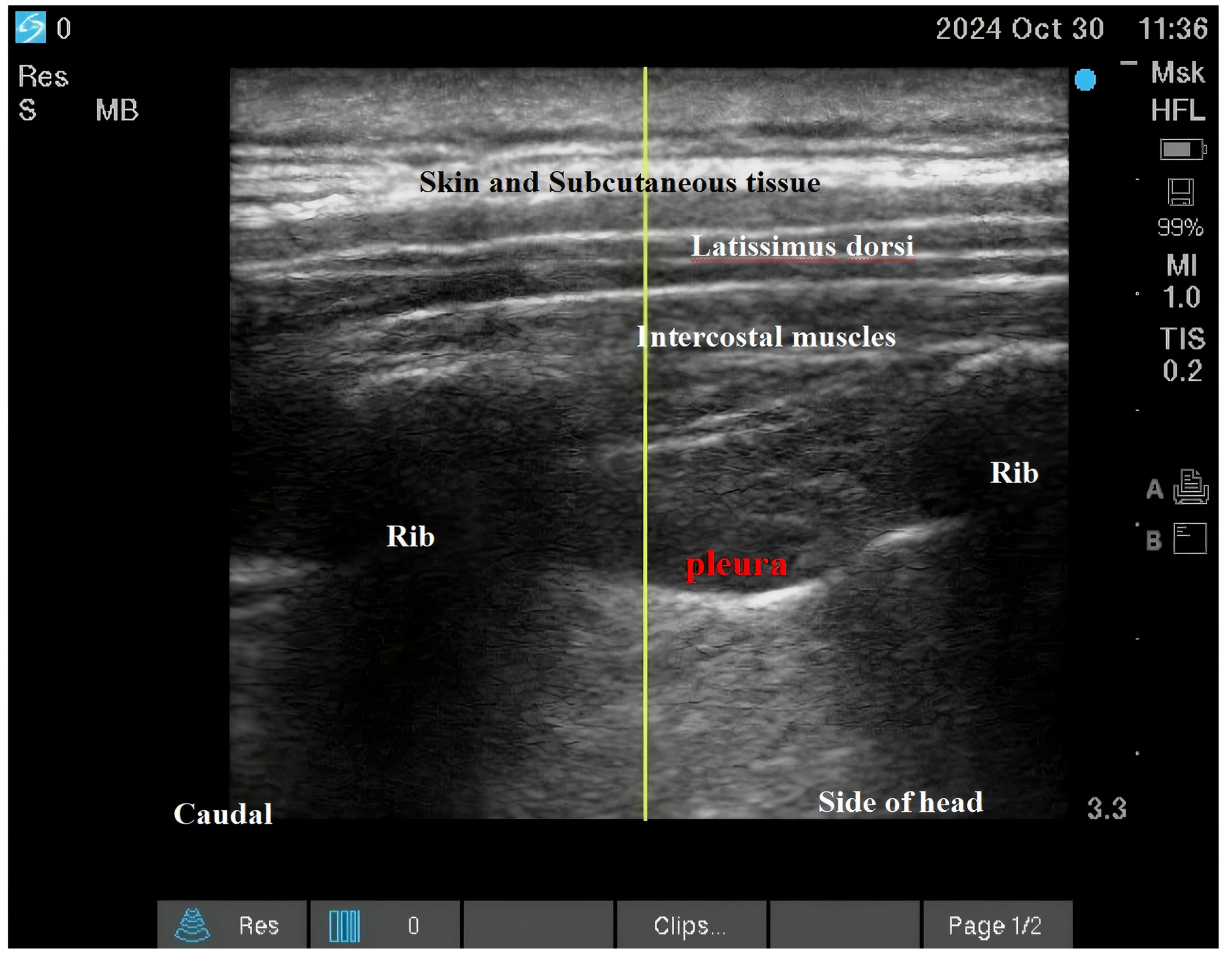
Intercostal nerve block.

Both interventions were performed as descending intercostal nerve blocks at ribs 2–6 using a standardized ultrasound-guided technique (SonoSite X-Porte; 6–13 MHz linear transducer) by a single experienced anesthesiologist (100 prior blocks performed). Needle placement and local anesthetic spread were confirmed in both transverse and longitudinal views (See Figures 2; 3).

### 2.6 Postoperative pain management and assessment

A standardized multimodal analgesia protocol was implemented for all patients:

Preemptive analgesia: 30 min before surgical closure: intravenous sufentanil (5 μg) + flurbiprofen axetil (15 mg).

PCA protocol: Solution: sufentanil (100 μg) + flurbiprofen axetil (200 mg) + ondansetron (16 mg) in 100 mL normal saline. Parameters: bolus dose 2 mL (sufentanil 2 μg), lockout interval 15 min, no background infusion.

Rescue analgesia: intravenous morphine 5 mg if VAS ≥ 4 after 3 PCA demands within 45 min.

Restrictions: No oral analgesics permitted, no additional non-study analgesics allowed, No regional analgesia supplements.

Postoperative pain was systematically evaluated using the Visual Analog Scale (VAS; 0–10), where 0 represented “no pain” and 10 indicated “worst imaginable pain.” Assessments were conducted at predetermined intervals (6, 8, 12, 24, 48, and 72 h postoperatively) by the same trained research staff following a standardized protocol. Every time first resting state scoring, followed by the patient’s exercise state scoring after coughing. The patient was excluded if the surgical procedure extended beyond 6 h after the initial assessment. If patients were found to be asleep during the scheduled VAS evaluation, a default score of 3 was assigned to represent a moderate pain level at rest or at exercise.

### 2.7 Statistical analysis

The primary outcomes were resting pain scores and exercise pain scores during coughing assessed at 6, 8, 12, 24, 48, and 72 h post-intervention. The secondary outcomes included cumulative morphine consumption (mg), PCA demand frequency (presses), patient satisfaction scores, intraoperative remifentanil dosage (μg), postoperative hospital stay duration (hours).

Continuous variables were presented as mean±SD or median[IQR] based on distribution, count data were reported as n (%). Normally distributed data: Independent t-tests, non-normal data: Mann–Whitney U tests. Categorical data: Compared with *χ*^2^ or Fisher’s exact tests. All tests were two-tailed with *α* = 0.05.

## 3 Results

**Figure 4:**
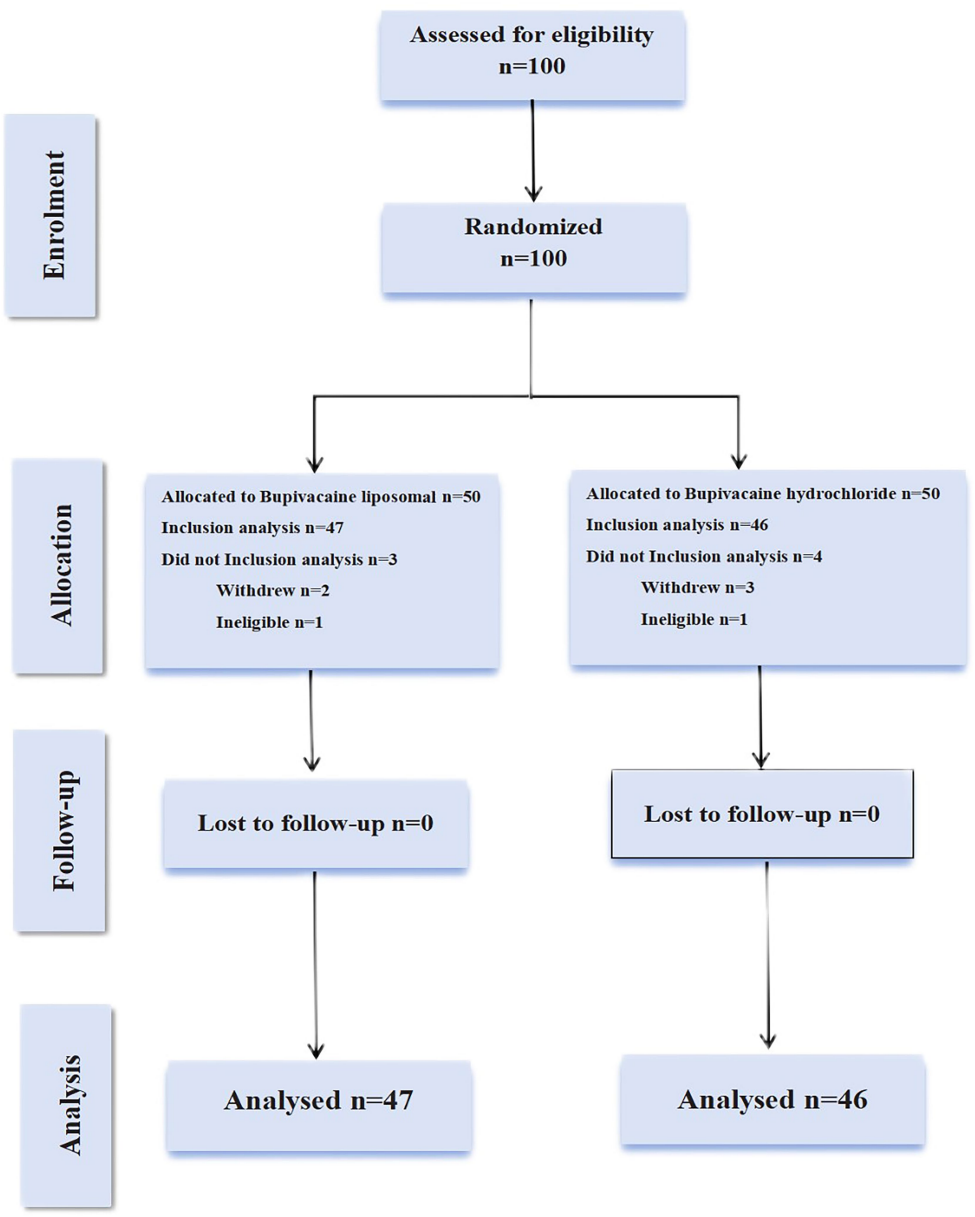
A detailed flow diagram of patient enrollment, randomization, and follow-up procedures. In the experimental group, one patient operated for more than 6 h, missing VAS pain score at 6 h, met the shedding criteria was removed, and the other two patients withdrew from the study. In the control group, one patient met the exclusion criteria due to the operational dose injection errors, and three others chose to drop out.

Between November 2, 2023 and September 26, 2024, we screened 100 patients for eligibility. During follow-up: LB group: 1 participant met exclusion criteria, while 2 withdrew consent. Control group: 1 participant was excluded, with 3 withdrawing. All attrition cases were documented with reasons (See Figure 4).

The study population (*N* = 93) had a mean age of 48.2 ± 9.6 years with balanced gender distribution [65 female (69.9%), 28 male (30.1%)]. Mean BMI was 23.0 ± 2.2 kg/m^2^. Surgical procedures included: segmentectomy: 63 cases (67.7%), wedge resection: 14 cases (15.1%), lobectomy: 16 cases (17.2%). Treatment allocation was nearly equal [LB: *n* = 47 (50.5%) vs. conventional bupivacaine: *n* = 46 (49.5%)]. Mean operative durations were LB group: 172.6 ± 49.0 min, control group: 187.3 ± 52.8 min. All measured baseline characteristics showed no significant intergroup differences (See Table 1).

**Table 1:** Baseline Characteristics of Study Population.

	Intervention (*N* = 47)*	Control (*N* = 46)#	All (*N* = 93)	*p*
Age(y)	47.7 ± 9.5	48.6 ± 9.8	48.2 ± 9.6	0.643
Sex, *n* (%)	Male 14 (29.8)	14 (30.4)	28 (30.1)	0.946
	Female 33 (70.2)	32 (69.4)	65 (69.9)	
BMI(kg/m^2^)	22.9 ± 2.0	23.1 ± 2.4	23.0 ± 2.2	0.656
Operation duration(min)	172.6 ± 49.0	187.3 ± 52.8	180.9 ± 51.0	0.232
Type of operation, *n* (%)				0.763
Segmentectomy	32 (68.1)	31 (67.4)	63 (67.7)	
Wedge resection	6 (12.8)	8 (17.4)	14 (15.1)	
Lobectomy	9 (19.1)	7 (15.2)	16 (17.2)	

BMI, body mass index (calculated as weight in kilograms divided by height in meters squared). Quantitative variables were expressed as mean ±SD. Categorical variables were presented as a number (n) and percentage (%). *Data were missing for 3 participants. # Data were missing for 4 participants.

### 3.1 Primary outcomes

**Figure 5:**
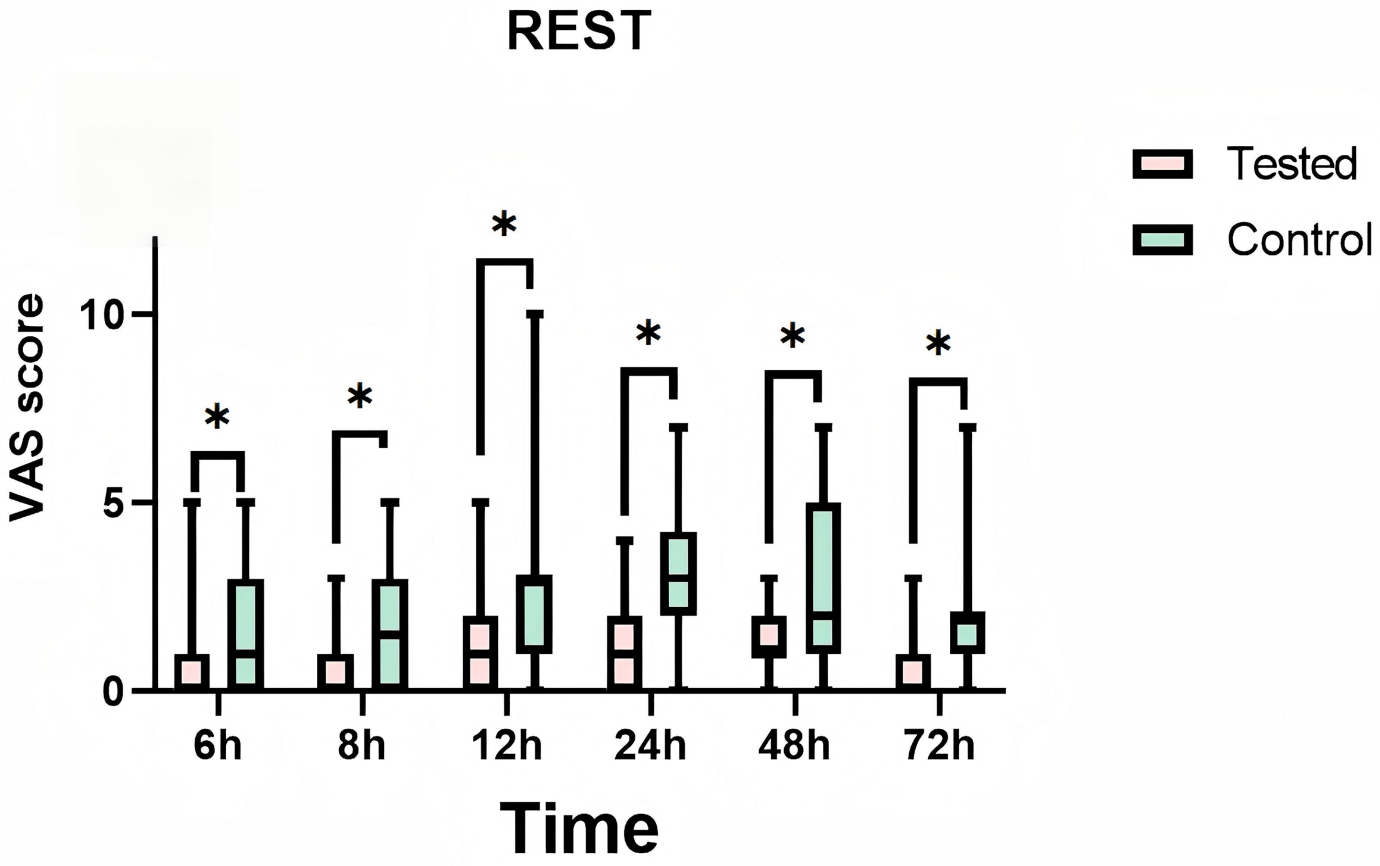
Mean pain visual analog scale (vas) scores at rest at each time point. Teated: Patients receive liposomal bupivacaine. Control: Patients receive bupivacaine hydrochloride. T1:6 h after administration, T2:8 h after administration, T3:12 h after administration, T4:24 h after administration, T5:48 h after administration, T6:72 h after administration. * is a statistically significant difference.

**Figure 6:**
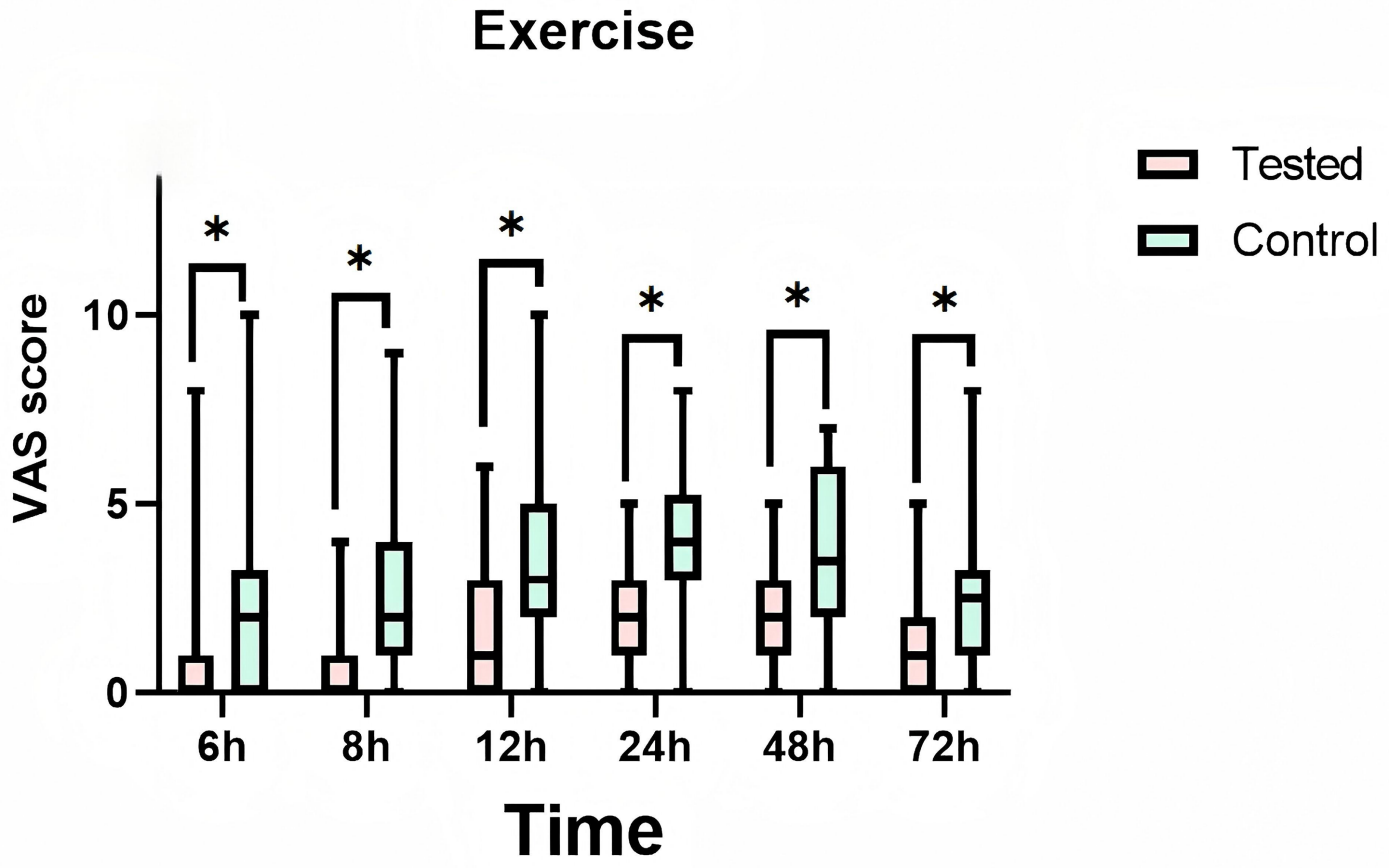
Mean pain visual analog scale (vas) scores at exercise at each time point. Treated: Patients receive liposomal bupivacaine. Control: Patients receive bupivacaine hydrochloride. T1:6 h after administration, T2:8 h after administration, T3:12 h after administration, T4:24 h after administration, T5:48 h after administration, T6:72 h after administration. * is a statistically significant difference.

During the 72-h observation period, a comparative analysis of VAS pain scores at rest and during exercise was conducted between the LB group and the bupivacaine hydrochloride group. The findings revealed that the LB group exhibited significantly lower pain scores than the control group. This reduction in pain was statistically significant (*p* < 0.01), indicating a clinically meaningful difference in analgesic efficacy between the two interventions (Table 2). Furthermore, Figure 5 and Figure 6 provide visual representations of the mean VAS pain scores at rest and at exercise, respectively, at each time illustrate the trends in mean VAS pain scores at rest and during exercise, respectively, across all assessed time points over the 72-h period.

**Table 2:** Mean postoperative Resting and exercise pain score.

	Intervention	Control	*p
6 h	0.0 [0.0, 1.0]	1.0 [0.0, 3.0]	<0.01
8 h	0.0 [0.0, 1.0]	1.5 [0.0, 3.0]	<0.01
12 h	1.0 [0.0, 2.0]	3.0 [1.0, 3.0]	<0.01
24 h	1.0 [0.0, 2.0]	3.0 [2.0, 4.3]	<0.01
48 h	1.0 [1.0, 2.0]	2.0 [1.0, 5.0]	<0.01
72 h	0.0 [0.0, 1.0]	2.0 [1.0, 2.0]	<0.01

	Intervention	Control	*p
6h	0.0[0.0,1.0]	2.0[0.0,3.3]	<0.01
8h	0.0[0.0,1.0]	2.0[1.0,4.0]	<0.01
12h	1.0[0.0,3.0]	3.0[2.0,5.0]	<0.01
24h	2.0[1.0,3.0]	4.0[3.0,5.3]	<0.01
48h	2.0[1.0,3.0]	3.5[2.0,6.0]	<0.01
72h	1.0[0.0,2.0]	2.5[1.0,3.3]	<0.01

The two sets of data did not conform to the normal distribution when they were tested for normality, so the data were expressed by median (interquartile range, IQR). VAS is a visual analogue scale; test group: liposomal bupivacaine group, control group: bupivacaine hydrochloride group;*p is a statistically significant difference.

### 3.2 Secondary outcomes

#### 3.2.1 Morphine consumption

The median morphine rescue consumption in both the LB and bupivacaine hydrochloride groups was zero (see Table 3). Although the nonparametric analysis revealed a statistically significant difference in rescue frequency between the two groups (*p* < 0.01), this finding lacked clinical relevance, as no meaningful intergroup disparity in morphine requirements was observed.

**Table 3:** secondary outcomes analysis.

	Intervention	Control	*p*
Morphine consumption(mg)	0.0 [0.0, 0.0]	0.0 [0.0, 5.0]	<0.01
Number of analgesic pump compressions(times)	11.0 [4.0, 20.0]	30.0 [15.8, 42.3]	<0.01
Remifentanil usage(μg)	1800.0 [1500.0, 2600.0]	2175.0 [1675.0, 3000.0]	0.088
Patient satisfaction(score)	9.0 [8.0, 10.0]	7.0 [5.8, 8.3]	<0.01
Length of hospital stay (day)	7.0 [6.0, 9.0]	8.0 [7.0, 9.0]	0.135

Data are presented as median (interquartile range, IQR).

#### 3.2.2 Number of analgesic pump compressions

A significant intergroup difference in analgesic pump utilization was observed during the 72-h postoperative period. The experimental group (liposomal bupivacaine) required markedly fewer pump activations (median = 11) compared to the control group (bupivacaine hydrochloride; median = 30). This difference was statistically significant (*p* < 0.01), indicating superior analgesic efficacy in the experimental group.

#### 3.2.3 Satisfaction of patients

Patient satisfaction analysis revealed statistically significant differences between the treatment groups (*p* < 0.01). The liposomal bupivacaine group demonstrated superior satisfaction outcomes (mean score = 9.0) compared to the bupivacaine hydrochloride group (mean score = 7.0), suggesting better patient-reported experiences with the experimental intervention.

#### 3.2.4 Length of hospitalization

The median hospitalization duration was 7.0 days for the LB group compared to 8.0 days for the control group. Statistical analysis revealed no significant intergroup difference in length of stay (*p* = 0.135), suggesting that the analgesic intervention had limited impact on this outcome measure. These findings imply that hospitalization duration also may be influenced by factors such as postoperative care protocols or individual patient recovery trajectories.

#### 3.2.5 Remifentanil usage

Intraoperative remifentanil consumption analysis revealed comparable usage between groups, with the liposomal bupivacaine group requiring a median of 1800.0 μg (IQR 1500.0–2600.0) compared to 2175.0 μg (IQR 1675.0–3000.0) in the control group (*p* = 0.088). This nonsignificant difference (*p* > 0.05) suggests that the experimental anesthetic technique did not substantially affect intraoperative opioid requirements.

### 3.3 Safety evaluation

Analysis of postoperative nausea and vomiting (PONV) incidence during the first 72 postoperative hours revealed a lower rate in the LB group (2.1%, 1/47) compared to the bupivacaine hydrochloride control group (8.7%, 4/46). However, this difference did not reach statistical significance (*p* = 0.345). The control group reported one instance of shoulder pain (2.2%), which was not observed in the LB group (0%). Neither group exhibited cutaneous pruritus, pulmonary complications, or cardiac function abnormalities (Table 4). These findings suggest that LB administration does not increase the risk of common postoperative complications relative to conventional bupivacaine hydrochloride, while potentially offering a numerically lower (though not statistically significant) incidence of PONV.

**Table 4:** Safety evaluation.

	Intervention *N* = 47	Control *N* = 46	*p*
nausea and vomiting [*n* (%)]	1 (0.0)	4 (8.7)	0.345
Itch of skin [*n* (%)]	0 (0.0)	0 (0.0)	None
Pulmonary complications [*n* (%)]	0 (0.0)	0 (0.0)	None
Abnormal cardiac function [*n* (%)]	0 (0.0)	0 (0.0)	None
Shoulder pain [*n* (%)]	0 (0.0)	1 (2.2)	0.495

Data are presented as *n* (%).

## 4 Discussion

The rising incidence of thoracic surgeries has underscored the critical need for effective postoperative analgesia. Clinical evidence reveals that in the immediate 24-h postoperative period, 20% of patients experience severe pain (VAS ≥ 7), while 75% report moderate-to-severe discomfort (VAS ≥ 4) (15). Of particular concern is the persistence of postsurgical pain, with 66% of patients continuing to experience moderate-to-severe pain at discharge and 9% reporting prolonged symptoms lasting up to 2 weeks postoperatively (16, 17). Chronic post-thoracotomy pain syndrome, often resulting from suboptimal acute pain management, may persist for years, significantly impairing quality of life and functional recovery (12). These findings emphasize that optimal perioperative pain control is not merely a comfort measure, but rather an essential component of surgical recovery that directly influences treatment outcomes.

Intercostal nerve blockade has become a preferred technique for postoperative pain management in thoracic surgery. A single injection can provide effective analgesia for up to 24 h postoperatively (18), offering significant advantages including: (1) superior pain control, (2) cost-effectiveness, (3) technical simplicity, and (4) an excellent safety profile. These characteristics collectively enhance patient satisfaction and comfort during recovery. The technique’s demonstrated efficacy in acute pain management and recovery facilitation has led to its widespread adoption, particularly in thoracoscopic procedures. Conventional local anesthetics (bupivacaine, lidocaine, ropivacaine) remain commonly employed, though their utility is constrained by the transient nature of single-injection analgesia (typically 4–8 h) (5). The advent of liposomal bupivacaine (DepoFoam™ technology) represents a significant pharmacological advancement (19). This formulation features multivesicular liposomal with aqueous chambers enclosed by phospholipid bilayers, enabling sustained drug release over 72 h. Its clinical applications extend to wound infiltration, periarticular injection, and peripheral nerve blocks (20). Despite the established use of bupivacaine hydrochloride for intercostal nerve blocks, robust comparative evidence regarding liposomal bupivacaine’s analgesic efficacy remains limited. This knowledge gap warrants further investigation through randomized controlled trials to establish optimal dosing protocols and duration of effect.

Our study demonstrates that LB provides superior postoperative analgesia compared to conventional bupivacaine hydrochloride in thoracoscopic surgery patients under general anesthesia. The LB group exhibited three key clinical benefits: (1) a 72-h extended analgesic duration, (2) a reduction in supplemental morphine requirements (*p* < 0.01), and (3) a decreased analgesic pump utilization (p < 0.01). This enhanced efficacy stems from LB’s unique pharmacokinetic profile - the multivesicular liposomal gradually release bupivacaine, maintaining therapeutic concentrations at intercostal nerve sites while minimizing peak plasma levels. Notably, this sustained release mechanism provided effective nociceptive blockade without increasing adverse events; we observed comparable safety profiles between LB and control groups, with no reported cardiotoxicity or pulmonary complications. Current evidence indicates LB contains only 5–8% free bupivacaine, with a bupivacaine hydrochloride concentration ranging from 0.06 to 0.1%, significantly below the 0.25% concentration used in conventional preparations (21, 22). Recent studies have further confirmed that LB extends analgesic duration, thereby reducing morphine requirements, decreasing opioid use, and minimizing reliance on general anesthesia (23–25). Additionally, several meta-analyses have demonstrated that LB reduces postoperative pain and opioid consumption in spine surgery (26, 27). This effect not only mitigates opioid-related adverse effects (e.g., nausea) but also underscores the clinical utility of LB within multimodal analgesia protocols, ultimately improving postoperative comfort and patient satisfaction (28–30). These findings support the potential of liposome-encapsulated bupivacaine to advance postoperative pain management strategies.

In this study, four cases of nausea/vomiting and one case of shoulder pain occurred in the bupivacaine hydrochloride group. Among the vomiting cases, two patients exhibited high analgesic pump usage: one required 50 pump activations and a morphine rescue dose, while the other activated the pump 22 times. These reactions were linked to excessive opioid administration. The remaining two vomiting patients each used the pump fewer than 15 times without requiring morphine; both were young women with a history of motion sickness, implicating heightened sensitivity to anesthetic agents. The shoulder pain case occurred in a patient with normal cardiac function but extreme opioid use (50 pump activations and three morphine rescues). We hypothesize that opioid-induced CNS depression—causing muscle relaxation and reduced pain perception—or potential intercostal nerve injury during block placement contributed to this symptom. The pain resolved after hot compress and massage therapy.

Our comprehensive safety assessment revealed no significant local tissue reactions at intercostal nerve block sites when using LB, in contrast to conventional bupivacaine hydrochloride. This finding suggests superior tissue compatibility of the liposomal formulation. The encapsulation of bupivacaine in liposomal likely attenuates its direct irritant effects, thereby minimizing local inflammatory responses compared to traditional formulations. Moreover, we observed no serious systemic adverse events associated with liposomal bupivacaine administration for intercostal nerve blocks. The controlled-release mechanism of liposomal bupivacaine maintains stable plasma concentrations, enhancing cardiac safety by reducing the risk of cardiovascular and central nervous system toxicity associated with peak bupivacaine plasma levels. These results strongly support the clinical safety profile of liposomal bupivacaine for regional analgesia. However, clinicians should remain cognizant that the active compound is still bupivacaine, which carries inherent cardiac toxicity risks. Strict adherence to recommended dosages is therefore crucial to prevent potential toxic reactions. In our study, we precisely administered 20 mL (266 mg) of liposomal bupivacaine per the manufacturer’s guidelines for intercostal nerve blocks. Consistent with current research findings from our team, none of the patients demonstrated signs of local anesthetic systemic toxicity (LAST), and the medication showed no adverse effects on surgical wound healing.

The timing of drug administration in this study was designed to leverage the well-established benefits of preemptive analgesia in acute pain management. As an evidence-based preventive strategy, preemptive analgesia has been widely shown to reduce both the incidence and intensity of postoperative acute pain. Current clinical guidelines strongly advocate for preemptive analgesia via analgesics or nerve blocks to mitigate acute postoperative pain (31). This approach not only reduces intraoperative opioid requirements but also stabilizes hemodynamic parameters during surgery, thereby enhancing both patient safety and comfort. Technically, we employed ultrasound-guided procedures to optimize drug delivery precision. Real-time ultrasound visualization of needle placement and local anesthetic spread minimized risks to adjacent anatomical structures while improving procedural safety. The administration timing was carefully selected based on the unique pharmacokinetics of liposomal bupivacaine. As a sustained-release formulation, liposomal bupivacaine demonstrates characteristically slow onset due to low extravesicular drug concentrations. Published data indicate its analgesic effects typically begin within 30 min post-administration, peak at 2–4 h, and last >72 h (21). To account for this profile, we performed awake nerve blocks prior to induction, ensuring therapeutic drug concentrations were achieved by surgical incision—a critical window for optimal preemptive analgesia.

### 4.1 Limitation

Several limitations should be acknowledged in this study. First, the single-blind design may introduce bias, particularly because pain assessment is inherently subjective. The operator-dependent pain assessments could have compromised the objectivity of the results. Second, the concurrent use of nonsteroidal anti-inflammatory drugs (NSAIDs) and variations in analgesic pump settings may have confounded pain score assessments. Although LB and conventional bupivacaine are not bioequivalent, the dose of any other forms of bupivacaine cannot be converted to the dose of LB, it should be set consistently in capacity to minimize the difference. Additionally, since pleural drains are a major pain source in VATS patients, the lack of comparison in drain indwelling duration between groups may have influenced block efficacy assessments. Postoperative pain can evolve from acute to chronic phases over months, yet our 72-h observation period precluded assessment of long-term outcomes, including chronic pain development and sustained analgesic efficacy. The current study was conducted with healthy young and middle-aged patients, results may not generalize to elderly, obese, multi-center settings. Future studies should extend follow-up durations and evaluate multiple dosing regimens in larger cohorts to determine optimal liposomal bupivacaine dosing and long-term outcomes.

## 5 Conclusion

In this randomized clinical trial, liposomal bupivacaine significantly improved postoperative recovery and pain management compared to bupivacaine hydrochloride in patients undergoing thoracoscopic surgery. Furthermore, LB significantly reduced opioid consumption, suggesting its potential as a viable alternative for postoperative pain control in patients undergoing minimally invasive lung resections. Further large-scale studies are warranted to confirm these findings.

## Data Availability

The original contributions presented in the study are included in the article/supplementary material, further inquiries can be directed to the corresponding author.
